# Heterogeneity in cost-effectiveness of lifestyle counseling for metabolic syndrome risk groups -primary care patients in Sweden

**DOI:** 10.1186/1478-7547-11-19

**Published:** 2013-08-28

**Authors:** Inna Feldman, Lennart Hellström, Pia Johansson

**Affiliations:** 1 Department of Women’s and Children’s Health, Uppsala University, Uppsala, Sweden; 2 Community Medicine, Uppsala County Council, Uppsala, Sweden; 3 Center of Public Health and health promotion, Kalmar County Council, Kalmar, Sweden; 4 Health economics, informatics and healthcare research, Stockholm County Council, Stockholm, Sweden

**Keywords:** Cost-effectiveness, Markov model, Metabolic syndrome, Lifestyle counseling, Primary care

## Abstract

**Background:**

Clinical trials have indicated that lifestyle interventions for patients with lifestyle-related cardiovascular and diabetes risk factors (the metabolic syndrome) are cost-effective. However, patient characteristics in primary care practice vary considerably, i.e. they exhibit heterogeneity in risk factors. The cost-effectiveness of lifestyle interventions is likely to differ over heterogeneous patient groups.

**Methods:**

Patients (62 men, 80 women) in the Kalmar Metabolic Syndrome Program (KMSP) in primary care (Kalmar regional healthcare area, Sweden) were divided into three groups reflecting different profiles of metabolic risk factors (low, middle and high risk) and gender. A Markov model was used to predict future cardiovascular disease and diabetes, including complications (until age 85 years or death), with health effects measured as QALYs and costs from a societal perspective in Euro (EUR) 2012, discounted 3%. Simulations with risk factor levels at start and at 12 months follow-up were performed for each group, with an assumed 4-year sustainability of intervention effects.

**Results:**

The program was estimated cost-saving for middle and high risk men, while the incremental cost *vs.* do-nothing varied between EUR 3,500 – 18,000 per QALY for other groups. There is heterogeneity in the cost-effectiveness over the risk groups but this does not affect the overall conclusion on the cost-effectiveness of the KMSP. Even the highest ICER (for high risk women) is considered moderately cost-effective in Sweden. The base case result was not sensitive to alternative data and methodology but considerably affected by sustainability assumptions. Alternative risk stratifications did not change the overall conclusion that KMSP is cost-effective. However, simple grouping with average risk factor levels over gender groups overestimate the cost-effectiveness.

**Conclusions:**

Lifestyle counseling to prevent metabolic diseases is cost-effective in Swedish standard primary care settings. The use of risk stratification in the cost-effectiveness analysis established that the program was cost-effective for all patient groups, even for those with very high levels of lifestyle-related risk factors for the metabolic syndrome diseases. Heterogeneity in the cost-effectiveness of lifestyle interventions in primary care patients is expected, and should be considered in health policy decisions.

## Background

The metabolic syndrome is a combination of medical disorders that increase the risk of developing cardiovascular disease and diabetes [1]. Many components of the metabolic syndrome are associated with lifestyles such as physical activity and food habits. Controlled preventive trials have shown that lifestyle intervention in primary care is effective in reducing metabolic syndrome risk factors, as well as the risks for diabetes [2-4]. Studies also indicate that lifestyle intervention is cost-effective for these patients, with reasonable costs per QALY (EUR 20,000–50,000 /QALY) [5-10].

Even though lifestyle modification is likely to affect the incidence of diabetes and cardiovascular disease, the effectiveness of lifestyle interventions probably differs over patient groups. This aspect becomes particularly important for interventions performed in standard primary care settings, as the patient characteristics and risk profiles of ordinary practice patients are likely to vary i.e. they demonstrate heterogeneity. Patient risk factor levels affect the risk for future disease but they might also affect the potential effects from the intervention; i.e. patients with higher risk factor levels might potentially experience larger decreases in disease risk after an intervention than patients with a lower risk level. The risk factor levels of patients might thus affect the effectiveness and hence the cost-effectiveness [11] of an intervention. The issue might be even more crucial for lifestyle interventions, as the potential benefits from the lifestyle modifications might differ according to individual ability to respond to the treatment as well as the individual baseline risk level. For example, the cost-effectiveness of lifestyle intervention has been shown to differ according to the participants’ BMI [12].

The influence of heterogeneity in patient characteristics on cost-effectiveness results is recommended to be investigated via subgroup analyses [13]. The importance of subgroup analyses in cost effectiveness analyses are also often underscored by decision-making agencies, such as the Swedish Dental and Pharmaceutical Benefits Agency [14] and the UK NICE [15]. Analyses that consider patient characteristics make it possible for decision-makers and clinicians to identify for which patient groups interventions are potentially cost-effective and therefore enable them to allocate resources in the optimal way [16].

The aim of this paper is to estimate the cost-effectiveness of a lifestyle program for metabolic syndrome patient with differing risk factor levels in ordinary primary care practice, and to explore the effects on cost-effectiveness from a risk factor grouping to account for patient heterogeneity. The program was called the Metabolic Syndrome Program and was implemented in the Kalmar regional healthcare area in Sweden. The cost-effectiveness analysis has previously been published in Swedish [17] but this paper seeks to elaborate on the long-term results over patient groups with different metabolic risk profiles.

## Methods

The Kalmar Metabolic Syndrome Program (KMSP) was implemented in the Kalmar regional healthcare area in the south-east of Sweden with approximately 63 000 inhabitants and a 6 per cent prevalence of diabetes type 2 (ages 16–84 years) [18]. The KMSP was a primary care-based intervention aiming to promote healthy lifestyles, in particular changes in dietary and physical activity habits, among patients with metabolic syndrome. Effectiveness was assessed via a before-and-after trial in which patient risk factor levels at the program start were compared with levels 12 months later. As the program was implemented in standard care circumstances, the patient follow-up was considered normal quality assurance and no ethical approval was thus necessary.

The cost-effectiveness analysis seeks to follow the Swedish recommendations [14] on health economic evaluations of pharmaceuticals, as medication frequently is the alternative to lifestyle intervention in primary care for the patient group considered. As recommended, costs are calculated from the healthcare as well as the societal perspective while health effects are expressed in QALYs (quality-adjusted life-years). Costs and QALYs are discounted 3 percent annually, conforming to the recommendations. Modeling is acceptable to estimate effects beyond follow-up, so long-term costs and health effects are simulated via a Markov model on the metabolic syndrome, incorporating cardiovascular disease (CVD) including stroke, and diabetes mellitus type 2 including diabetes-related complications. Costs were estimated in Swedish krona (SEK) in year 2004, inflated to reflect 2012 costs according to the Swedish consumer price index [19] and then transformed into Euro (EUR) (1 Euro 2012 = SEK 8.71). Cost-effectiveness is calculated in comparison with not implementing the program, which would mean that patients are only receiving standard care. As the program is regarded as a supplement to standard care, the incremental cost-effectiveness ratio is calculated with a so-called do-nothing alternative, which is assumed to imply zero costs and zero health effects.

### Patients

Half of the primary healthcare centers in the Kalmar regional healthcare area participated in the program, and implemented methods, to some extent adapted to local circumstances, of counseling and intervention during year 2004. Totally, 179 patients (80 men, 99 women) participated. The patient characteristics and risk factor levels (see Table 1) were collected from the primary healthcare records. To include a patient in the present analysis, four criteria had to be met: diagnosed with metabolic syndrome; registered as patient in a primary care centre at the start of the program; no increased drug treatment during the study period; and complete risk factor data at the start and end of the program. Of the 179 participants, 142 (62 men, 80 women; 79% of participants) met all inclusion criteria.

**Table 1:** Patient characteristics in the KMSP

	**Men (n = 62)**	**Women (n = 80)**
	**Mean (SD)**	**Mean (SD)**
**Age**	53 (11)	53 (10)
**Medical parameters**	Mean (SD)	Mean (SD)
Body mass index, BMI	32.5 (3.9)	32.3 (5.4)
Waist circumference (cm)	111.5 (9.8)	102.7 (12.1)
Fasting glucose (mmol/L)	6.4 (1.7)	6.3 (1.9)
Triglyceride (mmol/L)	2.4 (1.9)	1.9 (0.9)
HDL cholesterol (mmol/L)	1.1 (0.3)	1.4 (0.4)
Systolic blood pressure (mm Hg)	149 (18)	144 (19)
Diastolic blood pressure (mm Hg)	87 (11)	83 (9)
**Disease**	%	%
Previous myocardial infarction	13	2
Diagnosed diabetes	26	22
**Lifestyle**	%	%
Smoking	14	32
Physical activity > 30 min daily	51	52

### Risk groups

The primary care register data are used for classification of the patients into three clinically relevant risk groups (low, medium and high risk), as well as gender, which all affect the risk of contracting the metabolic syndrome diseases. The risk groups are defined according to the number of fulfilled criteria for metabolic syndrome [1]; central obesity (waist circumference), triglyceride (TG) level, HDL-cholesterol level, blood pressure (BP) level, and fasting glucose (FG) level, or previously diagnosed type 2 diabetes. A patient is regarded as in low risk if less than two criteria are met, medium risk if two criteria are met and at high risk if three or more criteria are met.

For each group, the average levels of risk factors at start and at follow-up (12 months) are calculated (see Table 2). Changes in levels are assumed due to the lifestyle program.

**Table 2:** Risk factor levels in risk groups and changes 12 months later

		**Men**			**Women**		
**Risk group**		**Low (n = 4)**	**Medium (n = 19)**	**High (n = 39)**	**Low (n = 37)**	**Medium (n = 28)**	**High (n = 15)**
Age							
	start	46	54	53	52	55	55
BMI¤							
	start	28.3	33.0	32.6	30.4	33.1	35.3
	12 months	27.2	32.6	32.2	29.6	32.0	34.7
	difference	-1.1^*^	-0.4^*^	-0.4^**^	-0.8^*^	-1.1^**^	-0.7^*^
Waist circumtance +							
	start	98	111	113	98	104	111
	12 months	93	109	110	94	101	104
	difference	-5^**^	-2^*^	-3^**^	-4^**^	-3^**^	-7^**^
Fasting glucose ¤,+							
	start	5.9	5.7	6.8	5.5	6.7	7.7
	12 months	6.1	5.6	6.5	5.4	6.5	7.1
	difference	+0.2	-0.1^*^	-0.3^*^	-0.1	-0.2^*^	-0.6^*^
Triglyceride +							
	start	1.1	1.4	3.0	1.4	2.2	2.6
	12 months	0.9	1.6	3.1	1.3	2.0	2.4
	difference	-0.2	-0.2^*^	-0.1	-0.1	-0.2	-0.2^*^
HDL cholesterol ,+							
	start	1.4	1.2	1.0	1.5	1.3	1.2
	12 months	1.6	1.3	1.1	1.6	1.4	1.3
	difference	+0.2^*^	+0.1^**^	+0.1^**^	+0.1^**^	+0.1^*^	+0.1^*^
Cholesterol¤							
	start	4.3	4.5	4.4	5.0	4.8	4.7
	12 months	4.2	4.3	4.3	4.9	5.0	4.4
	difference	-0.1^*^	-0.2^**^	-0.1^*^	-0.1	-0.2^*^	-0.3^*^
Systolic blood pressure ¤,+							
	start	140	148	150	137	147	154
	12 months	132	143	141	137	140	144
	difference	-8^*^	-5^*^	-9^**^	0	-7^*^	-10^**^
Diastolic blood pressure ,+							
	start	83	86	88	80	86	86
	12 months	80	85	84	78	84	81
	difference	-3	-1	-4^*^	-2^*^	-2^*^	-5^**^

^¤^ parameter in Markov model.

^+^ definition of metabolic syndrome.

** p < 0.01.

* p < 0.05.

Tests of statistical significance of differences over time within study groups were performed with a Wilcoxon signed rank test in SPSS (version 19).

### Program costs

The program costs are calculated in both a healthcare and societal perspective. The societal perspective includes all costs of implementing the project, i.e. the costs for the primary healthcare centres, for the patients and for other organizations, such as the local authorities that participated in some networking activities. The cost estimates have previously been reported in detail in Swedish [17].

The resource use was retrospectively identified and quantified from the program documentation and interviews with key persons, complemented by some standards (e.g. time for preparing lectures is assumed equal to the lecture time). The valuation of resources (see Table 3) was based on the costs of equipment for the Kalmar county council primary care Swedish wage statistics (Statistics Sweden) and some standards (e.g. costs for a meeting room). The costs for overheads and office equipment etc. were assumed to amount to 20% of the wage costs. Time costs for healthcare employees were valued in ten wage categories, while time costs for persons employed in other sectors were valued at the average Swedish wage. The time spent by the patients was valued at 35% of the average Swedish wage excluding wage taxes, a commonly used valuation of leisure time [20], as many program activities were performed during non-office hours. Costs for patients, according to program key persons’ information, include patient fees ( for some visits), time for individual and group meetings including traveling time, and time for physical activity performed within the program. Patient activities not organised by the program were not possible to include in the program cost calculation, implying an underestimate of the costs for the patients.

**Table 3:** **Valuation of resources**, **in Euro 2012**

**Resource**	**Unit**	**Value**
Wage, including wage taxes		
General practioneer	Hour	54
Higher official, politician	Hour	45
Qualified nurse	Hour	33
Primary care nurse	Hour	26
Public health official	Hour	25
Other primary healthcare personnel	Hour	24
Project coordinator	Hour	22
Assistant nurse	Hour	22
Average wage including wage taxes		
Public health office personnel	Hour	39
Primary care personnel	Hour	33
Sweden, for employed other sectors	Hour	54
Patient costs		
Time	Hour	6
Fees	Visit	11
Other resources		
Standard medical testing	Number	20
Photocopies	Number	0.26
Meeting room	Number	13
Fruit basket	Number	13
Coffee incl cake	20 people	26
Lunch	Number	9

### Model

To estimate the changes in future societal costs and health effects because of the program, a Markov microsimulation model on the metabolic syndrome was used. The model is described in detail in a technical report [21] (see supplement). In summary, the model incorporates the main diseases due to the metabolic syndrome; cardiovascular disease (CVD), including stroke, and diabetes mellitus type 2, including diabetes-related complications. All model cost data except medical treatment cost are taken from Swedish previously published studies, as well as the age-specific average quality-of life weights. All but one of the death risks are calculated from Swedish death registers, while the disease risks and the disease-related quality-of-life weights are taken from international studies. The disease-related costs seek to include costs to all sectors of society, such as costs for medical treatment, for institutional care, for pharmaceuticals, for informal care and other costs for patients and relatives, and productivity costs due to morbidity. The model was run as a microsimulation, with 10,000 repetitions. The termination age was 85 years, after which no further health effects or costs were accumulated. The cycle-length was one year. The model was programmed in DATA Pro Health Care (Treeage Software Inc, 2007).

The model simulations were based on average levels of some of the risk factors for each risk group at baseline and follow-up, as well as age and gender. To purge the estimates of age effects, the average age at baseline was used as the starting age for both simulations.

### Sustainability assumption

The analyses require an assumption about the duration of the program effects beyond the 12 months follow-up period; sustainability. We assumed that the patients would retain the risk factor levels measured at 12 months during another 12 months (i.e. during a total of 2 years), after which the changes would gradually decrease over the next 2 years, reaching the initial levels (i.e. those measured at the start) by the 5th year. This assumption is supported by some studies [22] , but previous modeling studies have used a number of different assumptions [10]. As the assumed sustainability is expected to affect the results considerably, the assumption is subjected to sensitivity analyses.

### Sensitivity analysis

In the model technical report [21] a large number of analyses on model parameters were reported, and, naturally, changes in model parameters lead to changed model estimates. In the present study, we report one multivariate sensitivity analysis of model parameters, only including previously available Swedish data, and one univariate methodological; discount rate 0 and 5 per cent. Sensitivity to the sustainability assumption was investigated for two alternatives; high sustainability, when intervention effects remain during the full lifetime of individuals, and low sustainability, when intervention effects only remain for 1 year and return to start levels in year 2 (i.e. no sustained effects after follow-up).

However, the most interesting analysis investigates the heterogeneity aspect. The base case analysis uses the average level over risk factor and gender groups to estimate the cost-effectiveness. Relevant alternatives are to refrain from the risk factor stratification, but to: 1). Divide the patients into gender groups with the average levels of the risk factors; 2). Divide the patients into gender groups but using the individual characteristics (risk factor levels and age) of the patients; and 3). No division into groups, but only individual characteristics (risk factor levels, age and gender) of the patients. The individual characteristics were obtained by stochastically selecting 10,000 patients among the participants, with their individual characteristics based on the actual patient data.

Finally, a probabilistic sensitivity analysis based on bootstraps of 1,000 replicates drawn 1,000 times from the 10,000 microsimulations was performed on men and women in the high risk group, depicted on the cost-effectiveness plane.

## Results

### Program costs

The total societal costs for the program as carried out at the primary care centres in Kalmar amounted to EUR 500,000, of which EUR 407,000 were paid by the healthcare organization. Costs for patients amounted to around EUR 78,800, while the costs for other organizations, mainly local authorities, totaled around EUR 19,700. These costs represent what a similar program would cost for county councils during a startup period of around 2 years on approximately 12 primary care centres for around 179 patients. The costs per patient amounted to EUR 2,810 in societal costs, and to EUR 2,300 in healthcare costs.

Note that some of the costs, such as those for personnel education and some program management, really should be regarded as one-time investments, and that the program would be considerably less costly if the program was to become implemented as standard care.

### Patient and model outcomes

The risk factor levels improved in all groups between start and follow-up at 12 months, except for fasting glucose in men in the low risk group (see Table 2), with most changes statistically significant at the 5% or 1% levels.

Table 4 reports the model estimates of future costs and health for the risk groups based on the risk factor levels at the start of KMSP and after 12 months. The improved risk factor levels for the risk groups result in slightly better health and lower future societal costs (see Table 4). The number of lost life-years (YLL) before the age of 85 years decreased in all risk groups by about 0.3 years. The expected number of QALYs increased in all groups, ranging from 0.05 to 0.14 QALYs, while future societal costs are estimated to decrease for all groups, by EUR 3,200-1,400.

**Table 4:** **Model estimated future health and costs in the risk groups at risk factor levels at the start of KMSP**, **at 12 months**, **and the difference (costs in Euro 2012)**

		**Men**			**Women**	
	**Low**	**Medium**	**High**	**Low**	**Medium**	**High**
Lost life-years (YLL)*						
start	6.31	6.06	9.62	4.17	7.64	9.49
12 months	6.05	5.75	9.32	3.94	7.36	9.15
difference	-0.26	-0.31	-0.30	-0.22	-0.28	-0.35
QALYs						
start	13.92	10.79	9.89	12.44	9.95	9.29
12 months	13.97	10.93	10.01	12.53	10.08	9.37
difference	0.05	0.14	0.12	0.09	0.13	0.08
Societal costs						
start	56443	52865	70948	48979	69191	71887
12 months	53998	49902	67727	46830	66850	70532
difference	-2446	-2963	-3221	-2149	-2341	-1354
Healthcare costs						
start	37314	37289	50093	35023	51911	53873
12 months	35616	35738	48232	33727	50612	53114
difference	-1698	-1551	-1862	-1295	-1299	-759

* Undiscounted.

However, the patients who participated in the KMSP are estimated by the model to be subjected to considerable risk for metabolic syndrome disease even after the intervention. They are estimated to experience a high number of lost life-years (between 4 and 9 years) in metabolic diseases before the age of 85, with estimated costs to society of about EUR50,000 – 70,000 per individual.

### Cost effectiveness analysis

The group-specific ICERs are calculated based on the average program costs of EUR2,810 (see Table 5). The net costs are negative for two of the male groups, the middle- and high risk, and low for remaining groups, with a maximum societal cost of EUR1,500 for high risk women. The costs per QALY for the low risk men and the low- and middle risk women are fairly low, below EUR8,000 and thus considered very cost-effective in Sweden [23]. For the high risk women the estimated societal costs per QALY amount to EUR18,000, which might be considered moderately cost-effective.

**Table 5:** **Costs per QALY per individual in the risk groups**, **societal and healthcare perspective, in Euro 2012**

		**Men**			**Women**	
	**QALYs**	**Net costs**	**Costs /QALY**	**QALYs**	**Net costs**	**Costs /QALY**
Societal perspective						
Low	0.05	364	7276	0.09	660	7337
Medium	0.14	<0	<0	0.13	469	3608
High	0.12	<0	<0	0.08	1455	18191
Healthcare perspective						
Low	0.05	561	11213	0.09	963	10698
Medium	0.14	707	5052	0.13	959	7379
High	0.12	397	3305	0.08	1499	18739

In a healthcare perspective, with program costs of EUR2,300, no net cost-savings are estimated for any groups, as the maximum cost-savings are estimated at EUR1,900 for the high risk men, but the ICERs are below EUR 20,000 for all groups.

The cost-effectiveness analysis thus indicates that there is heterogeneity in the cost-effectiveness over the risk groups, ranging from net savings to costs per QALY of EUR18,000. However, as all ICERs are considered cost-effective in Sweden, the heterogeneity in patient characteristics is unimportant for the overall conclusion on the cost-effectiveness of the KMSP.

### Sensitivity analyses

The base case result was not sensitive to the discount rate (see Table 6). Only including available Swedish model parameters result in higher ICERs, but they remain below the Swedish cost-effectiveness threshold for all groups. However, the sustainability assumptions influenced the ICERs to a very large extent. Low sustainability, i.e. no program effects after the 12 month follow-up, leads to increased net costs and decreased QALYs for men and women in all groups, but particularly for low risk women. This is to be expected; if middle-aged women’s risk factor levels are comparatively low and slight improvements only remain for one year it is implausible to expect long-term health benefits and cost decreases. For remaining groups, even the low sustainability assumption would result in moderate or low ICERs. The high sustainability assumption results in net cost savings for all patients groups except high risk women.

**Table 6:** Sensitivity analyses, in Euro 2012

		**Men**			**Women**	
	**QALY**	**Net costs**	**Costs/ QALY**	**QALYs**	**Net costs**	**Costs /QALY**
Base case						
Low	0.05	364	7276	0.09	660	7337
Medium	0.14	<0	<0	0.13	469	3608
High	0.12	<0	<0	0.08	1455	18191
Model parameters						
Low	0.16	3429	21433	0.18	700	3888
Medium	0.30	1919	6397	0.30	1922	6408
High	0.29	2123	7319	0.35	3974	11354
Discount rate, 0%						
Low	0.13	<0	<0	0.08	533	6663
Medium	0.37	<0	<0	0.16	<0	<0
High	0.38	<0	<0	0.14	185	1323
Discount rate, 5%						
Low	<0	1229	-	0.03	408	13611
Medium	0.12	<0	<0	0.08	689	8606
High	0.18	<0	<0	0.07	1269	18132
Sustainability, low						
Low	0.05	1200	24004	0.01	1517	151700
Medium	0.04	993	24822	0.08	910	11374
High	0.02	783	39146	0.05	1579	31587
Sustainability, high						
Low	0.44	<0	<0	0.28	<0	<0
Medium	0.43	<0	<0	0.45	<0	<0
High	0.57	<0	<0	0.49	1573	3210
Risk stratification						
1. Gender groups, average						
	0.19	<0	<0	0.11	<0	<0
2. Gender groups, individual characteristics						
	0.11	802	7290	0.07	953	13619
3. Individual characteristics						
	0.09	1261	14009			

The risk stratification did not alter the overall conclusion that KMSP is cost-effective. Among the alternative stratifications, the most simple division with average levels of risk factors over the two gender groups overestimates the cost-effectiveness, as the analysis indicates cost-savings for both genders. However, the two analyses that used the actual patient characteristics (analysis 2 and 3) indicate results that are very close to the base case analysis.

**Figure 1:**
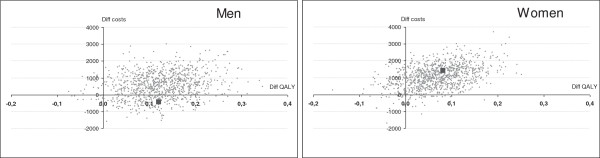
Probabilistic sensitivity analysis on the difference in costs (in Euro2012) and QALYs between at start and 12 months for high risk men and high risk women (large dot denotes base case difference).

The probabilistic sensitivity analysis, in Figure 1, reveals that there are marked differences between the distributions of ICERs between the high risk men and women. The uncertainty around the ICER is considerably larger among high risk men, but the women ICERs are more concentrated on the net cost increase quadrant. A large share of plots indicate net cost-savings among the men, and a vast majority low ICERs for both high risk groups.

## Discussion

This analysis reports that there is heterogeneity in the cost-effectiveness over the metabolic syndrome risk groups, due to the heterogeneity in patient characteristics, but this should not affect the overall conclusion on the cost-effectiveness of the KMSP. Cost-savings in the societal perspective was estimated for middle and high risk men, while the incremental cost vs do-nothing varied between EUR3,500 and 18,000 per QALY for other groups. Even the highest ICER (for high risk women) is moderately cost-effective in Sweden. In a healthcare perspective, no groups are estimated cost-saving, but the costs per QALY are low, below EUR20,000, for all groups.

Patients’ risk profile (low, middle, high) was defined as the number of metabolic syndrome risk factors (less than two, two, more than two). This graduation is very simple and does not require special medical investigations. This means that patients for whom treatment is most cost effective would be easily identified in routine clinical practice, which is an important consideration [16]. This is one of the most important results of this study. The risk stratification sensitivity analyses show that the risk factor grouping is appropriate, as the analyses using the individual patient characteristics gave very similar results. No risk stratification, i.e. using average risk factor levels (analysis 1), would tend to overestimate the cost-effectiveness. This has also been asserted by UK NICE; “Average cost-effectiveness can, therefore, mask important sources of heterogeneity which may be important to reflect in decision making…” ([15], p. 3). Furthermore, medical practitioners in standard primary care are fully aware of the fact that patients exhibit marked heterogeneity in risk factor levels, which could affect treatment results and thus cost-effectiveness. The aspect is increasingly discussed in terms of personalized medicine [24] or stratified medicine [25].

Many studies have shown that there are considerable differences in ICERs over patient groups, in such diverse areas as children otitis media [26], tuberculosis screening [27], assertive community treatment for homeless people [28] and phosphate binders in end-stage renal disease [29]. The Swedish health economic evaluation recommendations state that “The purpose of a health economic evaluation is to identify for which patient groups or indications a drug is cost-effective –it is never the medication itself which is cost-effective, rather the use of it” ( [14], p. 1). Nevertheless, the frequent use of subgroup analyses in clinical trials has been criticized [30]. There are also ethical issues in subgroup analyses, in particular if socio-demographic characteristics such as age, sex, race and social class, determine which patient groups will be offered treatment and if these characteristics are not directly related to the treatment effect [16].

One of the aims of the study is to seek to reflect Swedish primary care conditions, to enable decisions based on cost-effectiveness analyses in a Swedish ordinary praxis context. A formal trial design was not chosen, because the study sought to evaluate the program in ordinary primary care practice with the patient groups normally found there. The study design with a before-after comparison requires the assumption that all changes of lifestyles and ensuing changes in risk factors depend on KMSP. Possible changes in medical treatment were eliminated by use of the inclusion criteria that the patients had no increase in drug treatment during the study period. Most of the patients had had some kind of medication during a long period, so the positive effects from these medications had already been achieved before the measurement at the start was done. Some patients even decreased their medication during the program period. Note that these decreases in pharmaceutical costs were not included in the analysis, as the comparator the do-nothing alternative assumed no changes in costs for the standard treatment. This implies that the cost-effectiveness of the program is underestimated. An indication of the effectiveness of the KMSP is that all groups exhibited positive changes in risk factor levels at 12 months, mostly statistically significant. The effectiveness might also be underestimated, as we do not consider the preventive effects of lifestyle changes of other health problems, such as cancer or chronic pain, nor the short-term increases in quality-of-life during the project period included in the previous cost-effectiveness analysis of the project [17].

The result of the cost-effectiveness analysis seems to be in line with previous similar studies. The average costs per individual in the cost-effectiveness analysis of the Finnish DPS applied to a Swedish cohort [6] is around EUR26,000, which is comparable with the estimates for high risk women in our study. That study, however, only models cardiovascular disease. The US DPP study [5,31] reports average patient lifetime medical costs of around EUR50,000 (assuming year 2012 currency exchange rate), i.e. somewhat higher than our study estimates. Yet another comparison might be with the model simulations on US patients that fulfill the IDF criteria for metabolic syndrome [32], that resulted in 10 year healthcare costs of EUR37,000 for stroke patients and EUR18,000 for diabetes patients (assuming year 2012 exchange rate). These 10-year estimates seem more in line with our results.

Surprisingly, all risk factor groups were able to benefit from the lifestyle intervention. One might expect that lifestyle changes are more easily achieved among individuals that already lead comparatively healthy lives. However, all groups exhibit changes in risk factors, regardless of base line level. The risk factors that are most clearly related to changes in diet and physical activity, the weight and obesity parameters BMI and waist circumference, changed significantly in all risk groups. This indicates that even patients in the high risk group, with very high levels on risk factors, can change lifestyles if supported by an appropriate primary care program.

## Conclusions

Lifestyle interventions in primary care, like Kalmar Metabolic syndrome project, provide substantial health benefits at an attractive cost and, from the perspective of a fiscally prudent policymaker, represent the intervention of choice. The use of a risk stratification in the cost-effectiveness analysis established that the program was cost-effective for all patient groups, even for those with very high levels of lifestyle-related risk factors for the metabolic syndrome diseases.

## Competing interests

The authors declare that they have no competing interests.

## Authors’ contributions

IF planned the study, designed and performed the statistical analyses, including the risk factor groupings and the modeling, and drafted the manuscript. LH conceived of, planned the study, and collected the data. PJ conceived of, planned and designed the study, collected the cost data, advised on the modeling and the data analysis, and drafted the manuscript. All authors read and approved of the final manuscript.
